# Optogenetic control of protein binding using light-switchable nanobodies

**DOI:** 10.1038/s41467-020-17836-8

**Published:** 2020-08-13

**Authors:** Agnieszka A. Gil, César Carrasco-López, Liyuan Zhu, Evan M. Zhao, Pavithran T. Ravindran, Maxwell Z. Wilson, Alexander G. Goglia, José L. Avalos, Jared E. Toettcher

**Affiliations:** 1 Department of Molecular Biology, Princeton University, Princeton, NJ 08544 USA; 2 Department of Chemical and Biological Engineering, Princeton University, Princeton, NJ 08544 USA; 3 Department of Chemistry, Princeton University, Princeton, NJ 08544 USA; 4 Andlinger Center for Energy and the Environment, Princeton University, Princeton, NJ 08544 USA

**Keywords:** Optogenetics, Synthetic biology, Synthetic biology

## Abstract

A growing number of optogenetic tools have been developed to reversibly control binding between two engineered protein domains. In contrast, relatively few tools confer light-switchable binding to a generic target protein of interest. Such a capability would offer substantial advantages, enabling photoswitchable binding to endogenous target proteins in cells or light-based protein purification in vitro. Here, we report the development of opto-nanobodies (OptoNBs), a versatile class of chimeric photoswitchable proteins whose binding to proteins of interest can be enhanced or inhibited upon blue light illumination. We find that OptoNBs are suitable for a range of applications including reversibly binding to endogenous intracellular targets, modulating signaling pathway activity, and controlling binding to purified protein targets in vitro. This work represents a step towards programmable photoswitchable regulation of a wide variety of target proteins.

## Abstract (web summary)

The ability to regulate nanobody affinity with light would expand the applications toolbox for these reagents. Here the authors insert an optimised photoswitchable AsLOV2 domain into multiple nanobodies and demonstrate photoswitchable binding to fluorescent proteins and endogenous proteins in cells.

## Introduction

Nearly 20 years after the initial development of light-regulated transcription in yeast^1^ and light-gated ion channels in neuroscience^2^, optogenetics has been extended to almost every corner of cell biology. Optogenetic proteins are now available to control the fundamental currencies of protein heterodimerization^3–5^, homo-dimerization^6,7^, gene expression^1,8,9^, degradation^10^, nuclear-cytosolic translocation^11–14^, and even liquid–liquid protein phase separation^15–17^. These techniques have enabled a generation of precise perturbation studies to interrogate how the timing, spatial location, and identity of active proteins alter cellular and developmental processes.

Yet despite this growing suite of optogenetic tools, some applications have remained elusive. Light-triggered protein–protein interactions are typically induced between a light-sensitive protein and its natural binding partner derived from the original plant or cyanobacterial host (e.g., dimerization between PhyB/PIF6 or Cry2/CIB)^3,5,6^, or between engineered light-sensitive proteins and their fusions with special protein interactors to produce synthetic protein–protein interactions (e.g., binding of an engineered AsLOV2 variant to a PDZ or SSPB peptide or between Dronpa monomers)^4,7,18^. In contrast, achieving light-switchable binding to untagged proteins of interest has remained elusive. Recent work with chemical- and light-responsive dimerizers fused to split protein binders has achieved single-cycle off-to-on switching^19,20^, but reversibility and spatial control with these systems has not been realized. The ability to reversibly bind and release an untagged protein of interest in response to light would hold considerable promise for reversibly regulating endogenous signaling activity in living cells, developing biologics that can be precisely targeted in space and time, and enabling protein purification without affinity tags^21^.

Here, we present opto-nanobodies (OptoNBs): a class of engineered proteins capable of reversible, light-controlled binding against different untagged protein targets. Nanobodies, small binding proteins derived from the single variable domain of camelid antibodies, provide a versatile scaffold for obtaining high-affinity binding to a broad range of target epitopes and are functional in both intracellular and extracellular environments^22^. Our approach for obtaining photoswitchable nanobodies builds on recent pioneering work to insert a photoswitchable light–oxygen–voltage (LOV) domain into solvent-exposed loops on proteins of interest^23^. We identify loop insertion sites and LOV domain variants that trigger a light-inducible change in binding between four different nanobodies and three target proteins: EGFP, mCherry, and F-actin. We further demonstrate that OptoNBs can be used in cells for dynamic control over intracellular signaling and target binding with subcellular spatial precision and are functional in vitro for reversible control over protein binding. The OptoNB platform opens the door to developing light-switchable binders against a broad range of protein targets and may thus represent a first step toward an important class of photoswitchable biologics.

## Results

### Screening opto-nanobodies for photoswitchable binding

**Fig. 1:**
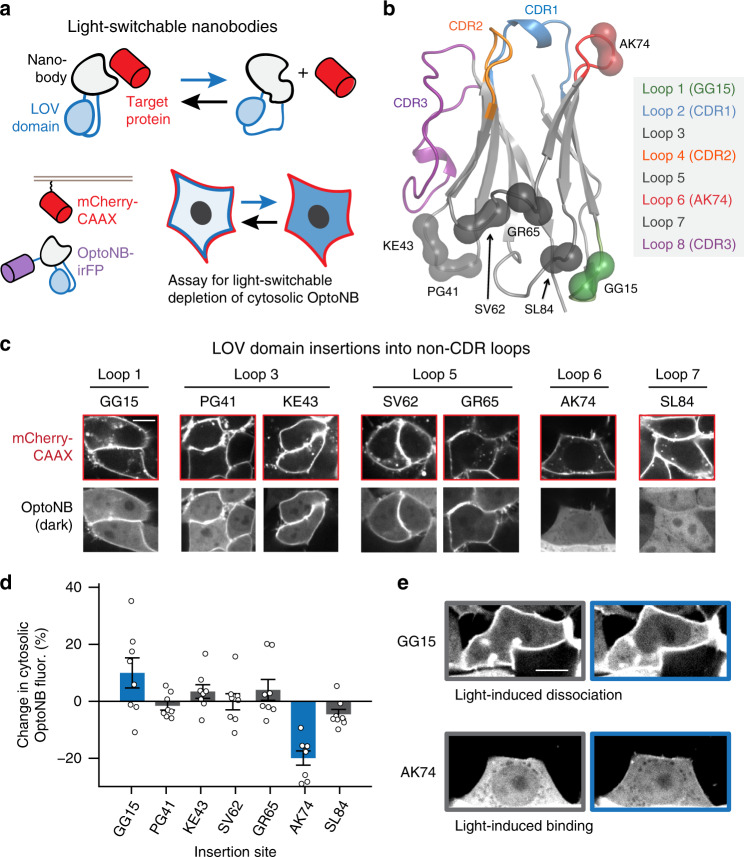
Initial screen for light-controllable opto-nanobodies (OptoNBs). **a** Schematic of approach. By insertion into a solvent-exposed turn or loop, the light-switchable AsLOV2 domain (blue) could modulate the conformation of a nanobody (gray), thus allosterically altering its ability to bind to a target protein (red). Cytosolic iRFP-fused OptoNBs were assayed for translocation to membrane-bound mCherry in the presence or absence of blue light. **b** Positions targeted for LOV domain insertions mapped onto the crystal structure of an anti-GFP minimizer nanobody (PDB ID: 3G9A). Spheres indicate the residues between which the LOV domain was inserted. Loops of interest and the hypervariable complementarity-determining regions (CDRs) are colored according to the legend. **c** Representative images for all LOV insertions. HEK293 cells expressing membrane-tethered mCherry (mCherry-CAAX) and cytosolic OptoNB-iRFP (OptoNB) are shown. **d** Quantification of light-induced change in cytosolic intensity for each OptoNB variant in (**c**). An increase in cytosolic OptoNB fluorescence corresponds to light-induced dissociation from membrane-bound mCherry, and vice versa for light-induced decrease in cytosolic iRFP. Error bars indicate mean ± SEM for *n* = 8 cells per variant. **e** Images before (gray box) and after (blue box) light stimulation for HEK293T cells expressing mCherry-CAAX and either of two OptoNB variants, LaM8-AK74 and LaM8-GG15, showing light-dependent changes in OptoNB localization. Images are representative of three replicate experiments. Scale bars: 10 μm. Source data are available as a Source data file.

Our strategy to engineer light-controlled nanobodies is based on pioneering work using ligand- or light-gated ‘hairpins’: –small domains that can be inserted in-frame into a solvent-exposed loop and that undergo a conformational change upon illumination or addition of a small molecule^23,24^. We reasoned that by inserting the light–oxygen–voltage-sensing domain from *Avena sativa* Phototropin 1 (AsLOV2) into  a solvent-exposed loop of a nanobody, it may be possible to allosterically alter the conformation of its binding surface, disrupting recognition of a target protein (Fig. 1a, upper panel)^21^. As a starting point, we focused on regulating binding between a model target protein, mCherry, and the LaM8 anti-mCherry nanobody^25^. We took a structure-based approach to identifying potential AsLOV2 insertion sites, testing all five conserved, solvent-exposed loops in the nanobody structure, excluding insertions in the hypervariable complementarity-determining regions (CDRs) (Fig. 1b).

We first set out to establish a cell-based assay for evaluating whether candidate opto-nanobodies exhibited light-switchable binding. Prior work has shown that monitoring cytosol-to-membrane translocation is a fast and sensitive method to characterize light-switchable binding (Fig. 1a, lower panel), revealing changes in protein localization for a diverse set of heterodimerization pairs and affinities^3,5,18^. We thus generated a set of HEK293 cell lines expressing a membrane-tagged mCherry target protein (mCherry-CAAX) and one of 10 candidate OptoNBs that were each fused on their C-terminus to an infrared fluorescent protein (OptoNB-iRFP) (Fig. 1a, lower panel). We then imaged each cell line to determine the OptoNB’s subcellular localization in the presence or absence of 450 nm blue light (Fig. 1c).

The initial screen yielded diverse results for different nanobody-LOV fusions. We observed colocalization between the nanobody and membrane-bound mCherry for LOV domains inserted into four out of five non-CDR loops (Loops 1, 3, 5, and 6) suggesting that nanobodies were broadly tolerant of LOV domain insertion without severe structural perturbation. Moreover, we observed some light-switchable redistribution between cytosol and membrane for two OptoNB fusions: those targeting the GG15 and AK74 insertion sites on Loops 1 and 6 of the nanobody (we name insertion sites based on the nanobody’s flanking amino acids and the position number of insertion, so AK74 corresponds to the sequence …-Ala-AsLOV2-Lys-… with AsLOV2 inserted after Ala74). Interestingly, light stimulation of these two sites triggered opposite effects on nanobody-mCherry binding: light-induced dissociation in the case of GG15 and binding in the case of AK74 (Fig. 1d, e). This set of chimeras with opposite response to light was surprising, as prior studies that took advantage of LOV insertion reported only light-induced disruption of protein function, which was explained by the increased flexibility of the light-stimulated state disrupting the fusion protein’s active state^23,26^. In contrast, our data on the AK74 chimera suggests that the AsLOV2 dark state can also disrupt function, which is restored upon blue light illumination. We constructed a second round of cell lines to test more insertion sites in Loops 1 and 6 where we obtained additional hits (positions 15–17 and 72–77) (Supplementary Fig. 1a). We observed similar light-induced changes at one additional site within each loop (GS16 and DN72), demonstrating that multiple sites within a single loop can be used for photoswitchable binding control (Supplementary Fig. 1b).

### An optimized LOV domain improves OptoNB function

Our initial screen also revealed that, for some OptoNBs (GG15, NA73, and MG77), light unexpectedly triggered nuclear export (Supplementary Fig. 1c, d). Nuclear export was observed even in cells that did not express membrane-bound mCherry; furthermore, it was quickly reversed in the dark for the GG15 and MG77 variants but was irreversible for the NA73 variant. We thus set out to improve the performance of our initial OptoNBs in two ways: eliminating undesired nuclear-cytosolic translocation of the nanobody and achieving a larger change in binding between dark and illuminated conditions.

**Fig. 2:**
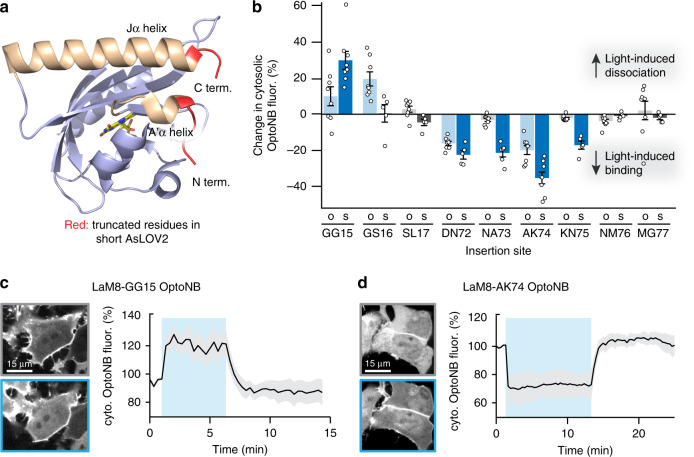
An optimized short-LOV domain for OptoNB engineering. **a** AsLOV2 crystal structure (PDB ID: 2V0U) indicating amino acids removed (red) to generate the optimized short AsLOV2 (408–543) for nanobody insertions. **b** Comparison of photoswitchable OptoNB binding in original AsLOV2 (‘o’) and short AsLOV2 (‘s’) for 9 insertion sites near the original two hits, GG15 and AK74. Blue bars indicate a light-induced change; gray bars indicate no photosensitive response. Error bars indicate the mean + SEM of the light-induced change in cytosolic intensity for *n* = 8 cells per variant. **c**, **d** Light-induced membrane/cytosol translocation in HEK293T cells for the LaM8-AK74 (in **c**) and LaM8-GG15 (in **d**) OptoNBs. The percent change in cytosolic intensity from the original, dark-equilibrated value is shown. Curves and shaded regions indicate mean ± SD for *n* = 10 cells per variant. Source data are available as a Source data file.

We hypothesized that the light-induced nuclear/cytosolic translocation might arise due to light-triggered exposure of a nuclear export sequence (NES), as has been engineered in prior AsLOV2-based optogenetic tools^12,14^. Indeed, amino acid sequence analysis revealed a canonical NES (LxxxLxxLxL, where x is any amino acid and L is a hydrophobic amino acid that is often leucine) spanning the junction between the C-terminal Jα helix and nanobody for the GG15 and MG77 insertion sites (Supplementary Fig. 1e). We did not observe a canonical NES for the NA73 variant, suggesting a different mechanism underlies its irreversible nuclear export. We thus sought to truncate residues from the LOV domain’s C-terminal junction to eliminate undesired NES activity. We also reasoned that truncating amino acids from the nanobody-AsLOV2 junctions may have an additional benefit, enabling tighter conformational coupling between the LOV domain and nanobody. A close examination of the crystal structure of AsLOV2 (PDB ID: 2V0U) suggests that removing linker residues at both the N and C termini of the AsLOV2 domain could more tightly couple it to the nanobody (Fig. 2a).

Based on this rationale, we constructed a ‘short LOV’ (sLOV) domain, comprising residues 408–543 of *Avena sativa* Phototropin 1 (versus residues 404–546 in Fig. 1 and 404–547 in ref. ^23^), and re-screened insertion sites near our two initial hits (Fig. 2b; Supplementary Note 1). We no longer observed light-dependent nuclear export in any sLOV insertions, consistent with the role of the C-terminal NES in this phenomenon. In addition, light-induced binding changes were enhanced in 5 of 6 cases (GG15, DN72, NA73, AK74, and KN75) compared with the original AsLOV2 constructs (Fig. 2b). We confirmed that light-switchable target binding could be reversibly toggled on and off for both light- and dark-inducible sLOV-containing OptoNB variants by measuring localization in cycles of darkness and blue light illumination (Fig. 2c, d and Supplementary Movies 1 and 2). These results demonstrate that variation in LOV domain’s C-terminal sequence can eliminate undesired nuclear/cytosolic translocation, and that truncating linker residues between the core LOV domain and its nanobody fusion partner can be useful for generating opto-nanobodies with enhanced photoswitchable binding.

### Developing OptoNBs for multiple scaffolds and targets

**Fig. 3:**
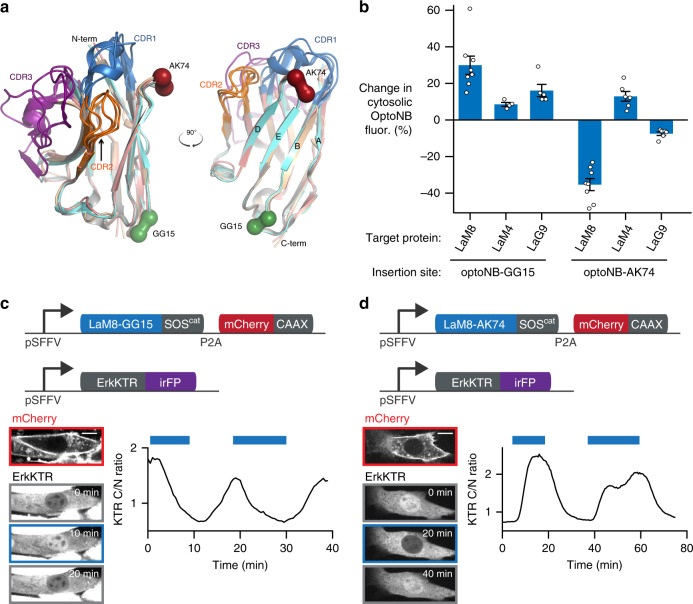
OptoNBs can be deployed against multiple targets and used to control intracellular signaling. **a** Superimposed nanobody structures with diverse binding modes: the anti-GFP minimizer nanobody in gray (PDB ID: 3G9A), anti-EGFR 7D12 in blue (PDB ID: 4KRL), anti-VGLUT Nb9 in beige (PDB ID: 5OCL), anti-Gelsolin Nb11 in pink (PDB ID: 4S10), and anti-CD38 MU551 in green (PDB ID: 5F1O). The CDRs, GG15 position, and AK74 position are highlighted in colors as indicated. **b** Light-induced translocation from nucleus to cytosol of LaM8, LaM4, and LaG9 OptoNBs with LOV insertion at GG15 or AK74. The change in cytosolic intensity from the original, dark-equilibrated value is shown. Error bars indicate mean ± SEM for *n* = 8, 4, 5, 8, 6, and 6 cells, respectively. **c**, **d** NIH3T3 cell lines harboring OptoNB-controlled Ras/Erk pathway activity using LaM8-GG15 (in **c**) and LaM8-AK74 (in **d**). Upper diagrams show lentiviral constructs expressed in each cell: membrane-localized mCherry-CAAX, OptoNB-SOS^cat^, and a live-cell biosensor of Erk activity (ErkKTR-iRFP). Lower diagrams indicate mCherry and ErkKTR expression and localization for representative cells. Curves show the cytosolic-to-nuclear ratio of ErkKTR for a representative cell when pulsed with blue light (blue bars). Images are representative of three replicate experiments. Scale bars: 10 μm. Source data are available as a Source data file.

Our initial OptoNB designs were in the context of a single binding pair: the LaM8 nanobody and its mCherry-binding partner. We next sought to test whether this light-induced binding or dissociation might also be found for other OptoNB scaffolds and regulate binding to additional targets. Both the AK74 and GG15 insertion sites of our LaM8 OptoNB are located in regions distinct from the hypervariable complementarity-determining regions (CDRs) and are conserved among nanobodies, including the higher-affinity mCherry nanobody, and an anti-EGFP nanobody LaG9 (Figs. 1b and 3a)^25^. We thus hypothesized that similar effects would be elicited upon LOV domain insertion in the same sites of each nanobody.

We generated sLOV insertions at the GG15 and AK74 equivalent positions in a second anti-mCherry nanobody (LaM4) and an anti-GFP nanobody (LaG9), fusing a C-terminal iRFP to each for tracking their localization in cells. We then co-transduced cells with a candidate OptoNB and membrane-localized mCherry or EGFP and monitored cytosolic OptoNB levels during cycles of blue light illumination or darkness (Fig. 3b). Insertion at the GG15 position led to consistent light-inducible dissociation in all three nanobodies, although both LaM4 and LaG9 exhibited weaker dissociation than LaM8, possibly due to the high affinity of these nanobodies to their targets leading to substantial binding in both the lit and dark states (reported dissociation constants of 0.18 and 3.5 nM, respectively, versus 63 nM for LaM8)^25^. In contrast, AK74 insertion led to highly variable results across all three nanobodies, with light-induced binding in the cases of LaM8 and LaG9, but light-triggered dissociation for LaM4. These observations are consistent with a model where light-induced destabilization of LOV domain contacts are more likely to disrupt protein function upon light stimulation than to restore it^23^, suggesting that the light-induced binding we observed in LaM8-AK74 may be a relatively unusual occurrence. We also compared the extent of cytosolic translocation from our OptoNBs to a gold-standard LOV-based heterodimerization system, the iLID-SSPB system, and found that LaM8-AK74 switches on a comparable scale to existing optogenetic tools in cells (~40% vs 60% changes in cytosolic intensity between light and dark, respectively) (Supplementary Fig. 2a).

Our comparisons between multiple OptoNBs also match intuition from available nanobody:target crystal structures^27–30^. Loop 6 lies relatively close to the target-binding surface of the nanobody (Fig. 3a, Supplementary Fig. 2b). Therefore, a LOV domain inserted in this loop might sterically interfere with target binding, which in some OptoNBs may predominantly occur when the LOV domain is in the dark-state conformation, and in others when it is in the lit state. This hypothesis is consistent with our observations (Fig. 3b). In contrast, Loop 1 is located far from the CDRs that comprise the binding surface for most nanobody:target interactions (Fig. 3a, Supplementary Fig. 2b). This suggests that the effect of light-triggered conformational changes of LOV domains inserted in Loop 1 are always allosteric in nature, in which the extended conformations of the lit state are more likely to cause disruptions to the target-binding surface of the nanobody domain than the dark conformation, again matching our observations (Fig. 3b).

During preparation of our study, a crystal structure of the LaM4 nanobody in complex with mCherry was deposited in the Protein Data Bank (PDB ID: 6IR1; Supplementary Fig. 3), providing a structure that we can directly compare to our optogenetic binding data. In this structure, LaM4 binds its target through CDR1 and CDR3, bringing mCherry close to the TK74 insertion site while still leaving space for a LOV domain to be inserted without clashing; in contrast, GG15 lays far from the binding interface. These observations further reinforce our model where Loop 6 insertion leads to allosteric light-induced dissociation for LaM4 but is close enough to the CDRs to exert steric effects in a target-specific manner.

### Coupling OptoNBs to Ras/Erk signaling in cells

Because our initial screen directly assayed for photoswitchable binding in mammalian cells, we reasoned that it should be possible to apply OptoNBs for light-based control over cellular functions without any further modification or optimization. To demonstrate this capability, we set out to construct an OptoNB-based variant of the OptoSOS optogenetic tool^31^. In this system, membrane localization of the catalytic domain of SOS (SOS^cat^) is used to trigger Ras activity^32^, activation of the Erk mitogen activated protein kinase cascade, and cellular responses including cell proliferation and differentiation. Light-induced signaling can also be easily visualized within minutes using the fluorescent Erk kinase translocation reporter (ErkKTR), a synthetic substrate that is exported from nucleus to cytosol within minutes of phosphorylation by active Erk^33^.

To reversibly trigger OptoSOS activation using nanobody-target binding, we generated NIH3T3 cell lines expressing an OptoNB-SOS^cat^ fusion protein (LaM8-GG15-SOS^cat^ or LaM8-AK74-SOS^cat^), membrane-localized mCherry (mCherry-CAAX) and an infrared fluorescent ErkKTR (ErkKTR-iRFP) (Fig. 3c, d top panels). We found that Erk activity could be rapidly toggled on and off with each OptoNB variant (Fig. 3c, d and Supplementary Movies 3 and 4). As expected from our initial protein-binding results, Erk signaling has opposite responses depending on which OptoNB is used to recruit SOS^cat^ to the membrane, with light-induced inactivation in LaM8-GG15 OptoSOS cells (Fig. 3c) and light-induced activation in LaM8-AK74 OptoSOS cells (Fig. 3d). These results demonstrate that OptoNBs can indeed be deployed in cells to manipulate cell signaling, at least in the context of the Ras/Erk kinase cascade. The high degree of amplification thought to exist in multi-step kinase cascades may be one reason that even modest changes in binding can have a large effect on pathway activity^34^.

### OptoNBs regulate protein binding in vitro

In addition to controlling intracellular protein–protein interactions, light-controlled nanobodies could be useful in vitro for a variety of applications, including as extracellular reagents to modulate receptor-level responses^35^, and the ability to decorate light-switchable binders in biochemical purification columns to separate unmodified target proteins based on light stimuli^36^. We thus set out to characterize the light-dependent performance of OptoNBs in vitro in a variety of assays: size exclusion chromatography, protein binding to OptoNB-coated agarose beads, and bio-layer interferometry-based measurement of OptoNB-protein-binding kinetics.

**Fig. 4:**
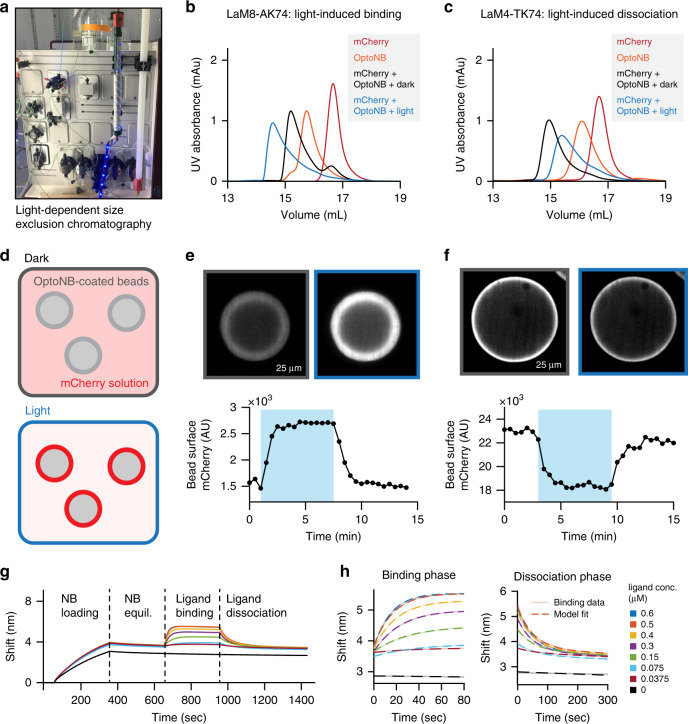
In vitro characterization of OptoNB binding. **a** Size exclusion chromatography (SEC) for light-dependent protein separation. The column was wrapped with 450 nm blue LEDs to allow for direct illumination of the protein during the SEC run, or in aluminum foil to keep it in darkness. **b**, **c** SEC elution profile for LaM8-AK74 (in **b**) and LaM4 TK74 (in **c**) in light conditions; for additional runs in the light and dark see Supplementary Fig. 4a, b. Free mCherry, free OptoNB, and dark- and light-incubated OptoNB/mCherry mixtures are shown in the indicated curves. Shorter retention times indicate larger size and increased complex formation. **d** Schematic representing light-induced binding of mCherry to OptoNB-coated beads. His-tagged OptoNBs are immobilized on Ni-NTA agarose beads, while untagged mCherry is in the buffer solution surrounding the beads. A change in illumination conditions results in mCherry-OptoNB binding and a brighter bead surface in the mCherry channel. **e**, **f** Top panels: confocal mCherry images of beads coated with a mixture of His-tagged EGFP with His-tagged LaM8-AK74 in a 200:1 ratio (in **e**) or LaM8-GG15 in a 1000:1 ratio (in **f**). Beads were placed in 1 μM mCherry solution (in **e**) or 2 μM mCherry solution (in **f**). A 450 nm LED was toggled on and off (blue shading indicates LED illumination). Bottom panels: quantification of bead surface intensity during cycles of darkness and blue light illumination. Images are representative of three replicate experiments. **g** Representative bio-layer interferometry (BLI) traces for quantifying nanobody-protein binding and dissociation kinetics. Four phases indicate His-tagged OptoNB loading onto the Ni-NTA coated tips, equilibration in buffer, binding to different concentrations of soluble mCherry, and mCherry dissociation into buffer. **h** Raw data (solid lines) and best-fit traces simultaneously fit to simple mass-action kinetic binding model (dashed lines) are shown for the immobilized LaM8 nanobody binding to soluble mCherry at eight concentrations. Source data are available as a Source data file.

As a first test of their function in vitro, we sought to test whether purified OptoNBs and their binding partners could be differentially separated in light and darkness using size exclusion chromatography. We expressed and purified the light-inducible binder LaM8-AK74 and the dark-inducible binder LaM4 TK74 from *E. coli* (see Methods). We wrapped a Superdex 200 10/300 GE column with blue light-emitting diodes (LEDs; Fig. 4a) or with aluminum foil (to keep dark conditions), and flowed solutions containing OptoNB, mCherry, or both in a 1:1.2 molar ratio of NB to mCherry through the column (Fig. 4b, c and Supplementary Fig. 4a, b). We observed a strong light-dependent shift in retention time for both OptoNBs. In the case of LaM8-AK74, light-induced binding leads to a shorter retention time under illumination (Fig. 4b, blue curve), and a longer complex retention time as well as a peak of free mCherry in the dark (Fig. 4b, black curve; compare to red curve for free mCherry). LaM4 TK74 exhibits the converse response, with shorter retention in the dark and longer in the light, indicating light-induced dissociation as previously observed in cells (Fig. 4c). Nevertheless, we saw that under both light and dark conditions, the OptoNB peak exhibited a shorter retention time in the presence of mCherry than when the OptoNB was run alone (Fig. 4b, c, yellow curve), indicating an equilibrium between binding and dissociation that was shifted but not eliminated by the change in illumination conditions.

Many in vitro and extracellular applications of protein binders are based on interactions on surfaces (e.g., cell surface receptor binding; affinity-based purification using bead-tethered antibodies). We thus sought to test whether OptoNB-target interactions could be controlled on the surface of agarose beads (Fig. 4d). We purified His-tagged OptoNBs (His_6_-LaM8-AK74 and His_6_-LaM8-GG15), as well as His_6_-GFP and His_6_-mCherry from *E. coli* (see Methods). To obtain beads with different surface densities of immobilized OptoNBs, we incubated nickel-NTA-coated agarose beads with solutions containing different ratios of anti-mCherry His_6_-OptoNB and His_6_-GFP (where His_6_-GFP was used as a bead surface blocking agent). We incubated the OptoNB-labeled beads with soluble mCherry (His_6_-cleaved) and imaged mCherry fluorescence during cycles of blue light illumination (Fig. 4e, f and Supplementary Movies 5 and 6). We observed a light-dependent shift in surface mCherry fluorescence as expected for AK74- and GG15-based OptoNBs. However, the time required to saturate the beads with bound nanobody upon light activation depends strongly on the density of target protein on the beads (Supplementary Fig. 4c). Complete mCherry binding was achieved within 10 s when beads were labeled with a 0.5%:99.5% LaM8-AK74:GFP protein ratio. In contrast, beads labeled with 100% LaM8-AK74 were only gradually saturated with mCherry over ~1 h. This phenomenon is likely due to local depletion of mCherry near the illuminated bead’s surface at high labeling densities, a picture that is consistent with the approximately linear increase in surface mCherry observed at high OptoNB concentrations^37^. In sum, we find that OptoNBs exhibit light-switchable binding to their targets across a broad range of contexts, from the mammalian intracellular environment to surface- and solution-based binding in vitro.

### Measurement of lit- and dark-state OptoNB binding kinetics

We next set out to quantify OptoNB binding in the lit and dark states. Binding measurements in the dark state can be made by taking advantage of the C450V point mutation in AsLOV2 which prevents photoadduct formation, rendering AsLOV2-based optogenetic tools light-insensitive^38,39^. Although a variety of lit-state mutants have been characterized that destabilize docking of the C-terminal Jα and N-terminal A’α helices^40,41^, it is unclear whether these mutations fully mimic the lit state when these helices are constrained by being inserted into loops of a chimeric fusion, as in our OptoNBs. We thus turned to bio-layer interferometry (BLI), a method that is compatible with sample illumination—so wild-type, light-sensitive LOV domains could be used—and which can be used to quantify both binding kinetics and affinity.

We first expressed and purified His-tagged variants of the parental LaM8 nanobody, the LaM8-AK74 and LaM8-GG15 OptoNBs, and OptoNB mutants that are expected to be light-insensitive and locked in the dark state (C450V equivalent) or in the presumptive lit state (I532E A536E equivalents)^42^. For each BLI run, one of these nanobody variants was loaded onto Ni-NTA-coated sensors, equilibrated in buffer, exposed to varying concentrations of mCherry to measure the association phase, and finally washed to measure the dissociation phase (Fig. 4g). The binding and dissociation curves at all mCherry concentrations were simultaneously fit to a simple mass-action chemical kinetic model of binding and dissociation, from which estimates of *k*_on_, *k*_off_, and *K*_D_ and their associated confidence intervals were obtained (Fig. 4h, see Methods and Supplementary Note 2). The global fitting procedure was able to fit the data well in each case (Supplementary Fig. 5; see Methods).

The resulting kinetics and affinities are presented in Table 1. We measured an affinity of 260 nM for wild-type LaM8, which differed somewhat from the 63 nM affinity reported previously^25^, possibly due to differences in assay design and procedures used to fit binding curves. We found that the LaM8-GG15 OptoNB also exhibits sub-micromolar affinity for mCherry in its dark state (530 nM for LaM8-GG15^C450V^), which is weakened to 2.5–3.1 μM in the lit state. In contrast, the LaM8-AK74 variant exhibits weaker affinity for mCherry in its dark state (29 μM) than its lit state (4.1–7.4 μM), just as we had observed in cells and in vitro. All lit-state measurements agree closely between illuminated, photosensitive OptoNBs and lit-state mutants, suggesting that these mutants accurately reflect the nanobody’s lit state. Finally, we note that in each case, the affinity change upon illumination was explained primarily by changes in the dissociation rate *k*_off_, with little change in the association rate *k*_on_. This observation is consistent with a light-dependent change in the complementarity of the nanobody’s binding site for its target protein, decreasing overall affinity by shortening the residence time of the bound complex. In sum, we demonstrate that bio-layer interferometry can be used to obtain binding kinetics and affinities for native lit-state optogenetic tools, without relying on mutants that may not perfectly approximate this state for a particular application. Applied to our LaM8-GG15 and LaM8-AK74 OptoNBs, BLI reveals between a 3.9- and 7-fold change in binding affinity between lit and dark states in vitro.

**Table 1:** Binding measurements for lit- and dark-state OptoNBs.

Variant	Illumination	*k*_on_ (μM^−1^ s^−1^)	*k*_off_ (s^−1^)	*K*_D_ (μM)
LaM8	None	0.072 ± 0.004	0.019 ± 0.001	0.26 ± 0.02
LaM8-GG15^C450V^ (pseudo-dark)	None	0.039 ± 0.003	0.021 ± 0.001	0.53 ± 0.05
LaM8-GG15^I532E,A536E^ (pseudo-lit)	None	0.038 ± 0.003	0.12 ± 0.01	3.1 ± 0.3
LaM8-GG15	450 nm light	0.038 ± 0.003	0.096 ± 0.002	2.5 ± 0.2
LaM8-AK74^C450V^ (pseudo-dark)	None	0.018 ± 0.004	0.52 ± 0.01	29 ± 10
LaM8-AK74^I532E,A536E^ (pseudo-lit)	None	0.034 ± 0.001	0.14 ± 0.01	4.1 ± 0.2
LaM8-AK74	450 nm light	0.021 ± 0.001	0.16 ± 0.01	7.4 ± 0.5

*k*_on_, *k*_off_, and *K*_D_ estimates indicate best-fit ±95% confidence interval.

### An actin binding OptoNB with spatiotemporal control in cells

One major application of light-controlled nanobodies is to modulate binding and unbinding of endogenous protein targets in living cells. Recent efforts have demonstrated substantial progress toward this goal: a split-nanobody strategy has been shown to be efficacious against a variety of endogenous proteins, and chemical dimerization has been successfully applied to a microtubule-binding nanobody^19,20^. Nevertheless, neither of these current approaches are reversible, and chemical dimerization cannot easily be extended to subcellular spatial control. We thus sought to test whether our LOV domain insertion strategy could be successfully deployed against an endogenous target. We chose actin as a first target as it is abundantly expressed, exhibits well-defined spatial localization in virtually all cells, and because excellent nanobodies are commercially available for its labeling in living cells^43,44^.

We designed a set of 13 LOV insertion variants into a TagRFP-fused actin nanobody, including our previously-successful GG15 and AK74 variant equivalents with both long- and short-LOV insertions, as well as long-LOV insertions in all other non-CDR loops where we had previously observed nanobody binding (Fig. 1b, c): Loop 1 (position 16), Loop 3 (positions 40–44), Loop 5 (positions 62–66), and Loop 6 (positions 72–75). Each construct was transiently transfected into NIH3T3 fibroblasts and assayed for localization to the actin cytoskeleton. As a control, we also assayed the parental actin nanobody lacking a LOV domain insertion, which co-localized to endogenous actin in nanobody-transfected cells.

**Fig. 5:**
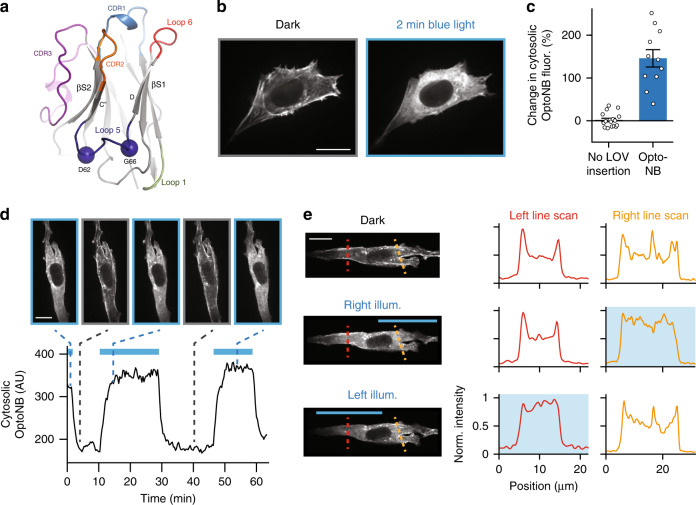
Development and characterization of an anti-actin OptoNB. **a** Position DG62-66 targeted for the LOV domain insertion into the α-actin nanobody and mapped onto the crystal structure of the anti-GFP minimizer nanobody (PDB ID: 3G9A). Residues D62 and G66 (blue spheres) are highlighted within Loop 5 (dark blue). CDRs, Loop 1, and Loop 6 are colored as previously described in Fig. 1. **b** Still frames showing dark and illuminated conditions for a cell expressing the actin OptoNB fused to TagRFP. **c** Light-induced translocation of actin OptoNBs. The light-induced change in cytosolic intensity from the original, dark-equilibrated value is shown. Error bars indicate means ± SEM for *n* = 17 and 11 cells, respectively. **d** Reversible actin OptoNB translocation to and from the cytoskeleton in a representative cell. Curve indicates the mean intensity in a cytosolic region in response to pulses of blue light (blue bars). Upper images show the cell at the indicated time points. **e** Spatial illumination leads to local nanobody unbinding. Left panels: representative images of a representative cell that was left un-illuminated or illuminated for 10 min on its left or right half. Dashed lines indicate positions of line scans for quantifying local enrichment along actin filaments. Right panels: quantification of actin OptoNB fluorescence along the line scans in dark, left, or right illumination. Scale bars: 20 μm. Source data are available as a Source data file.

Testing this library revealed nanobody-actin colocalization when LOV domains were inserted in either Loop 3 or 5 (Supplementary Table 1). Moreover, we observed exceptional light-switchable binding in a single Loop 5 variant in which the original LOV domain insertion was combined with truncation of three nanobody residues (63–65) (Fig. 5a; Supplementary Note 1), based on our intuition that the length and flexibility of Loop 5 might interfere with allosteric coupling. We found that this actin OptoNB undergoes light-induced dissociation, shifting from strong cortical and stress-fiber localization to diffuse, cytosolic localization upon illumination (Fig. 5b). Subsequent quantification across multiple cells revealed a >100% increase in cytosolic OptoNB intensity between dark and lit states (Fig. 5c). Binding and dissociation were rapid and reversible, reaching steady state within ~2 min as measured by quantifying changes in intensity of the cytosolic OptoNB pool, and showing no signs of degradation in performance after multiple cycles (Fig. 5d and Supplementary Movie 7).

Studies of cell polarization and migration in living cells are currently limited by lack of localized control over the cytoskeleton in specific regions of the cell, a difficulty that optogenetics is ideally poised to overcome^3,45^. As a first step toward direct control over the endogenous cytoskeleton, we tested whether local illumination could give rise to sharp patterns of nanobody-actin binding within individual cells. Subjecting single cells to local blue light stimuli revealed that nanobody binding was suppressed within the illuminated region, as seen previously with whole-cell illumination. Moving the light could also be used to toggle nanobody localization from one side of the cell to the other, labeling of cytoskeletal fibers in the un-illuminated side of the cell (Fig. 5e and Supplementary Movie 8). These differences were also apparent by quantifying OptoNB intensity through line scans of the cell, revealing loss of cortical enrichment at the cell edges during times of illumination (Fig. 5f). Together, these data demonstrate the construction of a light-switchable nanobody to an endogenous protein target with reversible, minutes-timescale temporal control and subcellular spatial targeting. This anti-actin OptoNB revealed a third loop in nanobodies in which insertion of a LOV domain can lead to light-switchable binding. Similar to the OptoNBs with LOV domain insertions in Loop 1 (at position GG15), the distance of Loop 5 from the epitope-binding surface of the nanobody suggests an allosteric mechanism of action.

## Discussion

Nanobodies are a class of proteins with high potential as biological reagents for cell and developmental biology^46,47^, biotechnology^36^, and therapeutic applications^35,48^ because of their small size, ease of expression in bacterial and eukaryotic cells, and high-affinity binding to a growing list of target proteins. Here we have shown that a simple strategy for constructing light-sensitive proteins—the insertion of a photoswitchable domain into a target protein—can be used to render nanobodies light-sensitive. We show that these OptoNBs can be produced against various targets, can exhibit either light-inducible or light-dissociable responses, and can be functionally coupled to cell signaling in cells and in binding assays in vitro.

The ability to control nanobody binding with light offers considerable promise for a variety of applications. Nanobodies can be raised against a broad range of target proteins through affinity maturation in immunized camelids, making it easy to envision OptoNBs whose binding can be toggled on and off from a broad range of endogenous targets. Nanobody binding can be inhibitory (e.g., by blocking an endogenous protein’s interactions with binding partners) or activating (e.g., by stabilizing an active conformation)^49^, opening the door to reversible, specific loss- or gain-of-function control. This is particularly intriguing, as most existing optogenetic tools regulate binding between two engineered domains without directly regulating endogenous, unmodified proteins. OptoNBs also have high potential for use as light-sensitive reagents outside of cells. Light-switchable binders could in principle be used to reversibly neutralize ligands or act as reversible agonists or antagonists on cell surface receptors on cells, tissues, or organisms that have not been genetically modified^35^; they could also enable separation of target proteins from complex mixtures without a need for affinity tags that may interfere with the target protein’s function^21,50^.

We identify four conserved loops where a LOV domain insertion can be tolerated without disrupting nanobody-target binding (Fig. 1c), and report cases where insertion in three of these loops (Loops 1, 5, and 6) result in photoswitchable binding. This flexibility may prove advantageous by providing opportunities to move the LOV domain insertion site to avoid steric interference with a particular target protein. Furthermore, we report two opto-nanobodies that can already serve as viable optogenetic tools for intracellular applications: the mCherry-binding LaM8-AK74, and the actin OptoNB. LaM8-AK74 reversibly binds mCherry in mammalian cells to a degree that is comparable to other high-quality optogenetic tools. Similarly, the actin OptoNB exhibits fast, light-switchable translocation that can be toggled on and off with subcellular spatial precision, demonstrating programmable control over binding to an endogenous unmodified protein target. We further demonstrate that localization changes can be harnessed for regulating intracellular processes by constructing an OptoNB-based variant of the OptoSOS system for controlling the Ras/Erk signaling pathway. It should be noted that mCherry is frequently used in intracellular labeling experiments, so LaM8-based OptoNBs could be immediately deployed as a ‘backwards-compatible’ strategy for altering the intracellular localization of these mCherry-tagged proteins in the cell lines or organisms where they have already been developed.

Nevertheless, improvements are likely to be needed before OptoNBs reach their full potential, especially in the case of in vitro applications. Our analysis of binding affinities in the lit and dark states indicates that our LaM8-based OptoNBs exhibit up to a 5.5-fold change between lit and dark states, spanning an overall 30-fold range of affinities between the tightest (610 nM for LaM8-GG15’s dark state) and weakest binders (19.9 μM for LaM8-AK74’s lit state). In addition, prior studies have designed engineered LOV domains and cognate peptides with up to 50-fold changes in affinity between lit and dark states^4^. These results indicate that there is still substantial room for improvement of photoswitchable OptoNB function.

One likely route to an improved OptoNB lies in optimizing the allosteric coupling between the LOV domain’s insertion site and nanobody’s target-binding surface. A larger change in binding affinity may be achieved by testing additional insertion sites, variations in the linker residues between nanobody and LOV domains, or by inserting light-switchable domain variants with increased dark-state stability^51^. We also note the apparent discrepancy between the large localization changes that we observed in cells (Fig. 2c, d) and the relatively small changes in affinity observed in our in vitro binding assay (Table 1). Nanobodies contain a disulfide bond that is non-essential for proper folding and target binding but may nonetheless alter the nanobody’s sensitivity to light-based allosteric control. It is possible that this disulfide bond is formed in our in vitro binding assays but reduced in intracellularly-expressed OptoNBs, leading to a difference in light-induced conformational changes in these two chemical environments, and suggesting that modifying these residues could serve as a good target for further optimization. Based on their existing capabilities and potential for improvement, OptoNBs hold considerable future promise for delivering programmable, light-controlled binding for broad range of applications inside and outside cells.

## Methods

### Plasmid construction

DNA containing LaM8, LaM4, and LaG9 was kindly gifted by Professor Kole Roybal (UCSF), and a plasmid encoding the anti-actin nanobody was purchased from Chromotek. All DNA was cloned using backbone PCR and inFusion (Clontech). pHR vectors were used for mammalian cell experiments and pBAD vectors were used for bacterial protein overexpression. AsLOV2 404–546 and AsLOV2 408–543 were ordered as gene blocks from IDT and used to insert into the nanobodies using inFusion (Clontech). The BFP-SSPB-SOScat-2A-PuroR-2A-iLID-CAAX plasmid was used to express the iLID/SSPB dimerization system as a single transgene^52^. All plasmids were cloned by amplifying appropriate sequences by PCR and performing assembly reactions using the inFusion kit (Clontech). All final plasmids were validated by sequencing (Genewiz). Stellar competent *E. coli* cells (Clontech) were transformed according to manufacturer’s instructions for all plasmid transformations. All OptoNB plasmids are available upon request from the authors or from the Addgene repository (accession number forthcoming).

### Lentivirus production and transduction

To produce lentiviral particles, HEK293T cells were plated on a 6- or 12-well plate and grown up to 40% confluency. At that point they were co-transfected with desired pHR plasmid and lentiviral packaging plasmids (pMD and CMV) using FuGENE HD (Promega). Virus was collected after ~48 h, filtered using a 0.45 mm filter, 2 μL of polybrene, and 20 mM HEPES were added to the viral particles. Either HEK293T or NIH3T3 cells were plated on a 6-well plate and infected with 200–500 μL of the virus at 40% confluency. Viral media was replaced by growth media 24 h post infection and imaging was done at least 48 h post the infection time. For iLID-SSPB translocation experiments, NIH3T3 cells were puromycin-selected after lentiviral transduction and a clonal cell line was established to limit cell-to-cell variability in expression. Media used for all cell culture maintenance contained DMEM, 10% FBS, penicillin, and streptomycin.

### Cell transient transfection

NIH3T3 cells were plated into 96-well plates at 40% confluency 2 days prior to imaging and 1 day prior to transfection. Twenty-four hours after plating cells, 300 ng of DNA encoding actin nanobody variant were transfected into cells. Transfection was done using Lipofectamine LTX reagent (Thermo Fischer Scientific) with 0.5 μL of PLUS reagent, 2.5 μL of LTX reagent, 20 μL of optiMEM. 10 μL of the mix was added into each well and then imaging was done 24 h after transfection occurred.

### Cell imaging

For imaging, 0.17-mm glass-bottomed, black-walled 96-well plates (In Vitro Scientific) were used. Glass was first treated with 10 μg/mL of fibronectin in PBS for 20 min. Cells were then plated and allowed to adhere onto the plate. Fifty microliters of mineral oil was added on top of the media prior to imaging to limit media evaporation. For RAS/Erk signaling experiments, cells were switched to starvation media prior to imaging (plain DMEM + 20 mM HEPES buffer, with no added serum). Cells were washed three times with starvation media and then equilibrated in starvation media for at least 3 h prior to imaging.

The mammalian cells were kept at 37 ˚C with 5% CO_2_ for the duration of all imaging experiments. Imaging was done using Nikon Eclipse Ti microscope with a Prior linear motorized stage, a Yokogawa CSU-X1 spinning disk, an Agilent laser line module containing 405, 488, 561, and 650 nm lasers, an iXon DU897 EMCCD camera, and a ×40 oil immersion objective lens. A 450 nm LED light source was used for photoexcitation with blue light, which was delivered through a Polygon400 digital micro-mirror device (DMD; Mightex Systems). For all LED illumination experiments we adjusted the LED power to a final value of ~1 mW/cm^2^ at the sample plane, as measured by a MQ-510 Quantum light meter with separate sensor (Apogee Instruments) using an equivalent blue LED light source placed above the sample. For all quantification of nuclear and cytosolic nanobody intensities, the background-subtracted intensities at the first timepoint (prior to light stimulation) and seventh timepoint (~2 min after light stimulation) were compared using the timecourse data included in the attached dataset.

### Protein expression

All proteins were expressed using pBAD N-His vector. Nanobody and opto-nanobody plasmids were transformed into Shuffle T7 Express *E. coli* cells (NEB) and EGFP and mCherry plasmids were transformed into One Shot Top 10 cells (Invitrogen). A single colony was used to inoculate a 10 mL 2x YT overnight culture supplemented with 200 μg/mL of Carbenicillin (Carb). The following day the culture was used to inoculate 0.5 L of 2x YT/Carb media that was shaken at 37 °C and 250 rpm until it reached an OD600 of ~1.0. Subsequently, the temperature was decreased to 20 °C and protein expression was induced by adding 0.2% Arabinose. The culture was shaken in the dark for ~18 h followed by harvesting the cells by centrifugation at 4 °C and 12,000 × *g*. If the protein was not purified right away, the pellets were stored at −80 °C.

For the purification, the 0.5 L cell pellet was thawed and resuspended in 25 mL of resuspension buffer (50 mM Tris pH 8.0, 150 mM NaCl) with 0.4 mM phenylmethanesulfphonylfluoride (PMSF) as well as a tablet of cOmplete Mini (Roche) and 14 μL of β-mercaptoethanol. Cells were lysed using a sonicator and the supernatant clarified by centrifugation at 250,000 × *g* for 1 h. Subsequently, FMN (0.25 mg/mL) was added to the supernatant with ~30 min incubation to ensure a homogenous distribution of the chromophore. Three to four milliliters of Ni-NTA superflow resin (Qiagen) were loaded onto a column and equilibrated with the resuspension buffer. The supernatant was loaded onto the column followed by 100 mL washes with resuspension buffer containing increasing concentrations of imidazole of 10, 20, and 30 mM. The protein was eluted at 250 mM imidazole and dialyzed overnight against resuspension buffer with the protein purity determined by SDS-PAGE. Protein concentrations were determined by recording the Abs_280_ and the following extinction coefficients for EGFP, mCherry, LaM8, and OptoLaM8NBs, 24,995, 34,380, 24,535, and 47,905 M^−1^ cm^−1^, respectively.

### Size exclusion chromatography

The size exclusion chromatography was performed on an AKTA Pure system (GE Healthcare) at 4 ˚C. The Superdex 200 Increase 16/300 GL column (GE Healthcare) was equilibrated with 50 mM Tris pH 8.0, 150 mM NaCl and this buffer was used for all subsequent SEC experiments. The purified proteins were assembled in 1:1.2 molar ratio of mCherry:nanobody or mCherry:OptoNB. The final volume of the proteins loaded onto the column was 50–200 μL, depending on the protein concentration. For experiments run in the dark, the lights were turned off, the chromatography refrigerator was covered in a black blanket, and the column was wrapped in aluminum foil. For experiments run in the light the column was wrapped with a blue LED strip with approximate intensity of 30 μmol/m^2^/s (Grainger). Before loading the proteins onto the column, the mixed samples were incubated for 20 min at room temperature, either in blue light or dark, according to the experiment that was being performed.

### Agarose bead imaging

Ni-NTA agarose resin (Qiagen) was first equilibrated with 50 mM Tris pH 8.0, 150 mM NaCl buffer. To competitively label the resin beads, solutions of 500 μL 1:10, 1:100, and 1:1000 nanobody:EGFP solution was loaded onto 200 μL of resin slurry with the excess protein washed away with the same buffer. Fifty microliters of 1 μM mCherry (with its His-tag cleaved off using TEV protease) was added onto 0.17-mm glass-bottomed black-walled 96-well plate (In Vitro Scientific). Two microliters of the nanobody bead slurry was added to the well with mCherry solution and incubated for at least an hour and up to overnight. The same microscope setup (imaging and blue light excitation) was used to image the beads as previously described for the cell imaging, except for the use of a ×20 air objective lens for the beads.

### Bio-layer interferometry measurements of binding kinetics

Measurements for the on rates (*k*_on_), off rates (*k*_off_), and affinity constants (*K*_D_) for LaM8, LaM8-AK74, and LaM8-GG15 nanobodies were performed on Octet RED96e instruments (ForteBio). Ni-NTA sensors (ForteBio) were first equilibrated in 50 mM Tris pH 8.0, 150 mM NaCl buffer for 10 min prior the measurement. Clear 96-well plates were used for the measurements and wells were filled with 200 μL of buffer or sample. During the experimental run the sensors were first immersed in a buffer to record the baseline, then switched to load the His-tagged nanobody onto the sensor and back into the buffer to remove unbound nanobody. To measure the association rate, the sensors were subsequently moved into a well with eight different concentrations of tag-less mCherry including a control with 0 mM mCherry. To measure the *k*_off_ the sensors were then moved into wells with a buffer and the dissociation rate was recorded. In order to measure binding kinetics of the light state, the lid to the Octet remained open during the measurement and a blue LED strip with approximate intensity of 30 μmol/m^2^/s was held above the 96-well plate keeping the protein in the light state for the duration of the experiment. The raw binding and unbinding data were simultaneously fit to models of binding and unbinding reactions using Eqs. (1) and (2):

1 $$y_{{\mathrm{bind}}}^i\left( t \right) = \left[ {a_{{\mathrm{on}}}^i\left( {1 - {\mathop{\rm{e}}\nolimits} ^{ - \left( {k_{{\mathrm{on}}}\left[ {{\mathrm{mCh}}} \right]_i + k_{{\mathrm{off}}}} \right)t}} \right) + b_{{\mathrm{on}}}^i} \right]{\mathrm{e}}^{ - k_{{\mathrm{leak}}}t}$$

2 $$y_{{\mathrm{unbind}}}^i\left( t \right) = \left[ {a_{{\mathrm{off}}}^i{\mathop{\rm{e}}\nolimits} ^{ - k_{{\mathrm{off}}}t} + b_{{\mathrm{off}}}^i} \right]{\mathrm{e}}^{ - k_{{\mathrm{leak}}}t}$$

This model incorporates the following dependent and independent variables:

$y_{{\mathrm{bind}}}^i\left( t \right)$ refers to the *i*th binding curve

$y_{{\mathrm{unbind}}}^i\left( t \right)$ the *i*th unbinding curve

[mCh]_*i*_ refers to the concentration of mCherry used for the *i*th binding curve

*t* is the time elapsed since the start of the binding/unbinding phase.

It includes the following parameters:

*k*_on_ is the on-rate (same across all binding and unbinding curves)

*k*_off_ is the off-rate (same across all binding and unbinding curves)

*k*_leak_ represents the slow unbinding of His-tagged OptoNB from the probe, leading to a gradual decay of signal

$a_{{\mathrm{on}}}^i$ is the total change in signal due to mCherry binding for the *i*th curve

$b_{{\mathrm{on}}}^i$ is the signal baseline during the binding phase

$a_{{\mathrm{off}}}^i$ is the total change in signal due to mCherry unbinding for the *i*th curve

$b_{{\mathrm{off}}}^i$ is the signal baseline during the unbinding phase.

The model thus contains 4*n + 3 parameters, where *n* is the number of distinct mCherry concentrations tested. Nonlinear fitting was performed using gradient descent using the MATLAB fmincon function. The MATLAB code used to perform the fits and calculate parameter confidence intervals is available on Github at https://github.com/toettchlab/Gil2020.

### Statistical methods

All data entries in Table 1 indicate the best-fit parameter value and 95% confidence interval obtained by globally fitting all binding data from a single nanobody variant to the mass-action kinetic binding model of Eqs. (1) and (2), which can lead to considerably better parameter estimates than performing separate fits for each dataset^53,54^. To compute the 95% confidence interval for *k*_on_, *k*_off_, and *K*_D_, parameter fitting was repeated starting from the optimum parameter values **p**_**opt**_ as an initial guess, except where a single parameter (one of *k*_on_, *k*_off_, or *K*_D_) was fixed to values over a 4-fold range above and below their best-fit value. The normalized chi-squared $\chi _N^2\left( {\mathbf{p}} \right)$ for each fitted parameter set **p** was then computed according to Eqs. (3) and (4), assuming data uncertainty *σ*_data_:

3 $$\chi ^2\left( {\mathbf{p}} \right) = {\mathop {\sum}\limits_{i = 1}^{n_{{\mathrm{data}}}} {\frac{{\left( {{\mathrm{data}}} \right)_i \,-\, \left( {{\mathrm{model}}} \right)_i}}{{\sigma _{{\mathrm{data}}}}}}}$$

4 $$\chi _N^2\left( {\mathbf{p}} \right) = \frac{{\chi ^2\left( {\mathbf{p}} \right)}}{{\chi ^2\left( {{\mathbf{p}}_{{\mathbf{opt}}}} \right)}} = \frac{{\mathop {\sum}\limits_{i = 1}^{n_{{\mathrm{data}}}} {\left( {{\mathrm{data}}} \right)_i \,-\, \left( {{\mathrm{model}}\left( {\mathbf{p}} \right)} \right)_i} }}{{\mathop {\sum}\limits_{i = 1}^{n_{{\mathrm{data}}}} {\left( {{\mathrm{data}}} \right)_i \,-\, \left( {{\mathrm{model}}\left( {{\mathbf{p}}_{{\mathbf{opt}}}} \right)} \right)_i} }}$$

The cutoff in chi-squared corresponding to the 95% confidence interval was obtained using the F distribution statistic in Eq. (6). Typical values were *n* = 35 parameters and *DF* ~ 2800 data points for a typical global fit to 8 BLI curves:

6 $$\chi _N^2\left( {\mathbf{p}} \right) = 1 + \left( {\frac{n}{{DF}}} \right)F_{0.05}\left( {DF,n} \right)$$

At the 95% confidence interval, the 95% cutoff of the F-statistic was approximately *F*_0.05_ = 1.43, yielding a confidence threshold of $\chi _N^2\left( {\mathbf{p}} \right) = 1.018$. The errors in Table 1 report the maximum change in parameter value such that the sum-of-squared error remained below the confidence threshold and thus represent an upper-bound on the 95% confidence interval.

### Reporting summary

Further information on research design is available in the Nature Research Reporting Summary linked to this article.

## Supplementary information

Supplementary Information

Description of Additional Supplementary Files

Supplementary Movie 1

Supplementary Movie 2

Supplementary Movie 3

Supplementary Movie 4

Supplementary Movie 5

Supplementary Movie 6

Supplementary Movie 7

Supplementary Movie 8

Reporting Summary

## Data Availability

The data that support the findings of this study are available from the corresponding author upon reasonable request. Raw data for Figs. 1d, 2b–d, 3b–d, 4c–f, 5c–e, Supplementary Figs. 1c, 2a, 4c, and Table 1 are provided as a Source data file.

## Source data

Source Data

## Code availability

All microscopy data was collected using NIS Elements version 4.4 (Nikon), quantified in ImageJ v1.52p, and analyzed using custom MATLAB scripts, which are available on Github at https://github.com/toettchlab/Gil2020.
