# Supplementary material for: Optogenetic control of protein binding using light-switchable nanobodies
Source: Nat Commun. 2020 Aug 13;11:4044. doi: 10.1038/s41467-020-17836-8 (PMC7426870; doi:10.1038/s41467-020-17836-8)
Supplement: Supplementary file 1 — Supplementary Information [file 41467_2020_17836_MOESM1_ESM.pdf]

Supplementary Materials for

Optogenetic control of protein binding using light-switchable nanobodies

Agnieszka A. Gil[1], César Carrasco-López[2], Liyuan Zhu[1], Evan M. Zhao[2], Pavithran T. Ravindran[1], Maxwell Z. Wilson[1], Alexander G. Goglia[1], José L. Avalos[1,2,3], Jared E. Toettcher[1,2]

Correspondence to: toettcher@princeton.edu, javalos@princeton.edu

This PDF file includes:

Supplementary Figures 1-5
Supplementary Table 1
Supplementary Note 1

Supplementary Figures and Legends

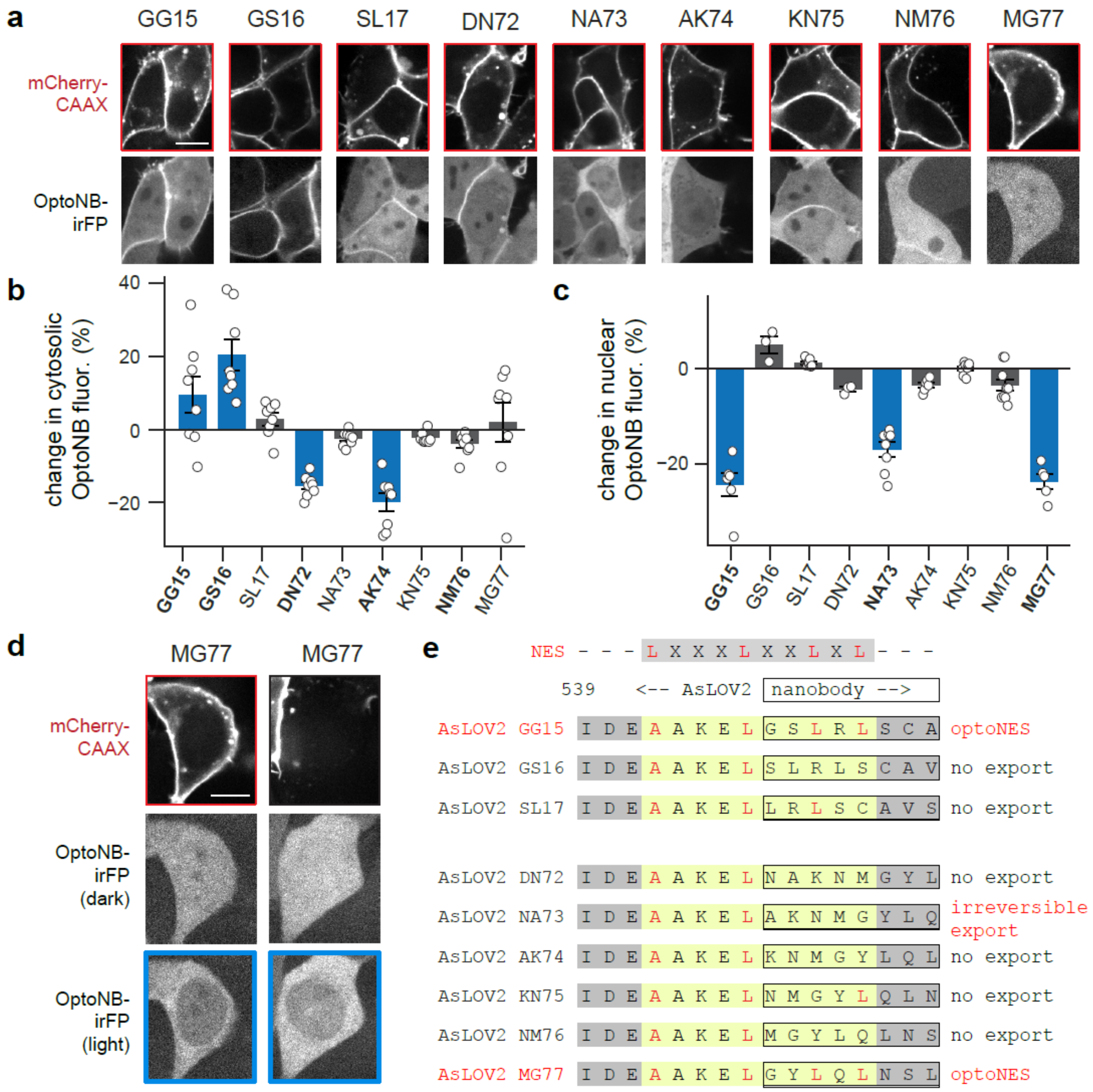

Supplementary Figure 1: Light-triggered intracellular translocation of initial OptoNBs. (**a**) OptoNB variants around GG15 and AK74 insertions. Upper: Membrane-localized mCherry-CAAX. Lower: Initial OptoNB-iRFP localization in the dark. Images are representative of two replicate experiments. (**b**) Light-induced change in cytosolic fluorescence of OptoNB variants around GG15 and AK74 insertions indicating light-induced dissociation of GG15 and GS16, and light-induced binding of DN72 and AK74 OptoNBs. Error bars indicate mean ± SEM for n=8 cells per variant. (**c**) Light-induced change in nuclear fluorescence of OptoNB in cells without mCherry CAAX membrane component. Reversible light-induced nuclear export is observed in GG15, and MG77, along with light-induced irreversible nuclear export in NA73. Error bars indicate mean ± SEM for n=6,3,8,3,8,6,8,10, and 5 cells, respectively. (**d**) Light-induced nuclear export in MG77 variant. Left panels: a cell expressing LaM8-MG77 and mCherry-CAAX; right panels show a cell expressing LaM8-MG77 only. Images representative of three replicate experiments. (**e**) A canonical nuclear export sequence (LxxxLxxLxL) is mapped onto the sequence at the C-terminal junction between AsLOV2 and the nanobody fusion for various OptoNB insertion sites. For all image panels, scale bars = 10 µm.

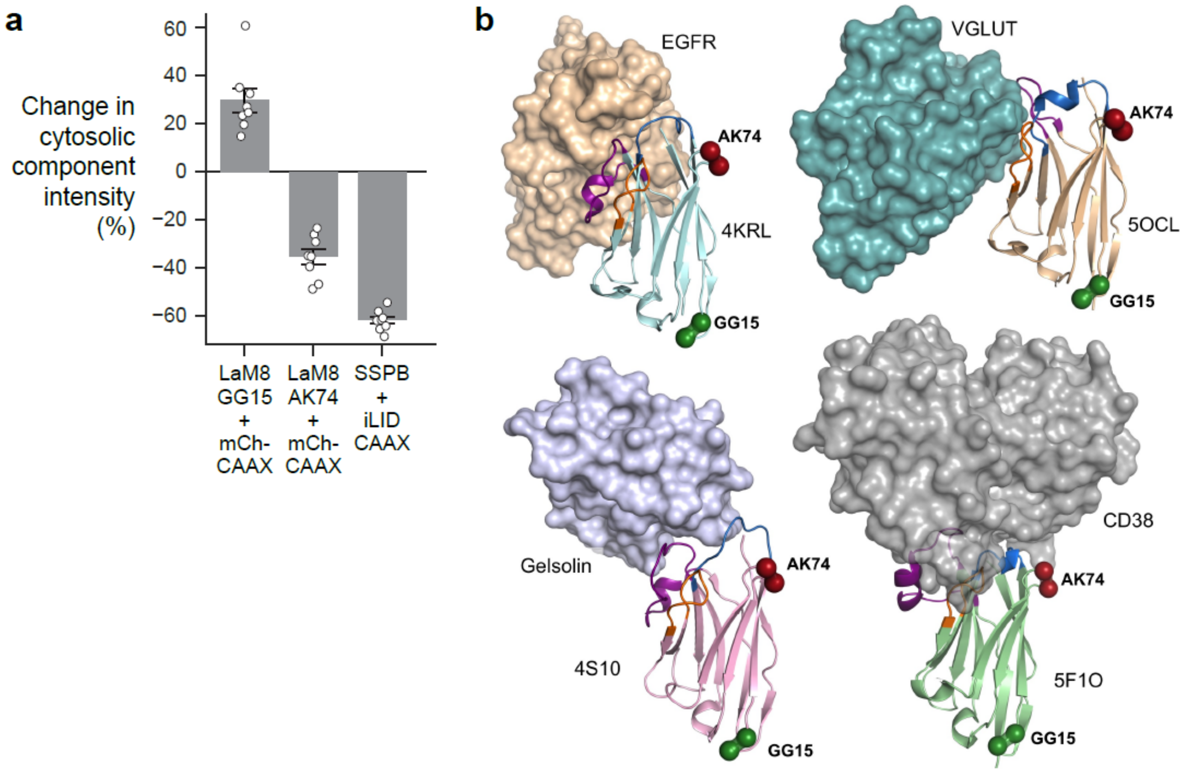

Supplementary Figure 2: (**a**) Comparison of light-induced translocation for the OptoNBs LaM8-GG15 and LaM8-AK74 with a gold-standard optogenetic system, the iLID-SSPB translocation system. Error bars show mean ± SEM for n=8 cells per variant. For iLID/SSPB translocation, cytosolic BFP fluorescence was measured for NIH3T3 cells with a stably integrated BFP-SSPB-SOScat-P2A-iLID-CAAX lentiviral construct. (**b**) Crystal structures of representative nanobodies in complex with their targets. The structures of nanobodies 7D12 (blue), Nb9 (beige), Nb11 (pink) and MU551 (green), are shown as cartoons. The structures of their corresponding target proteins – EGFR (beige), VGLT (teal), Gelsolin (light purple) and CD38 (gray) – are represented as surfaces. The corresponding PDB codes of each complex and the names of the protein targets are labeled, and the positions of the AK74 (red balls) and GG15 (green balls) insertion sites are indicated. CDRs are colored as previously described in **Fig 1**.

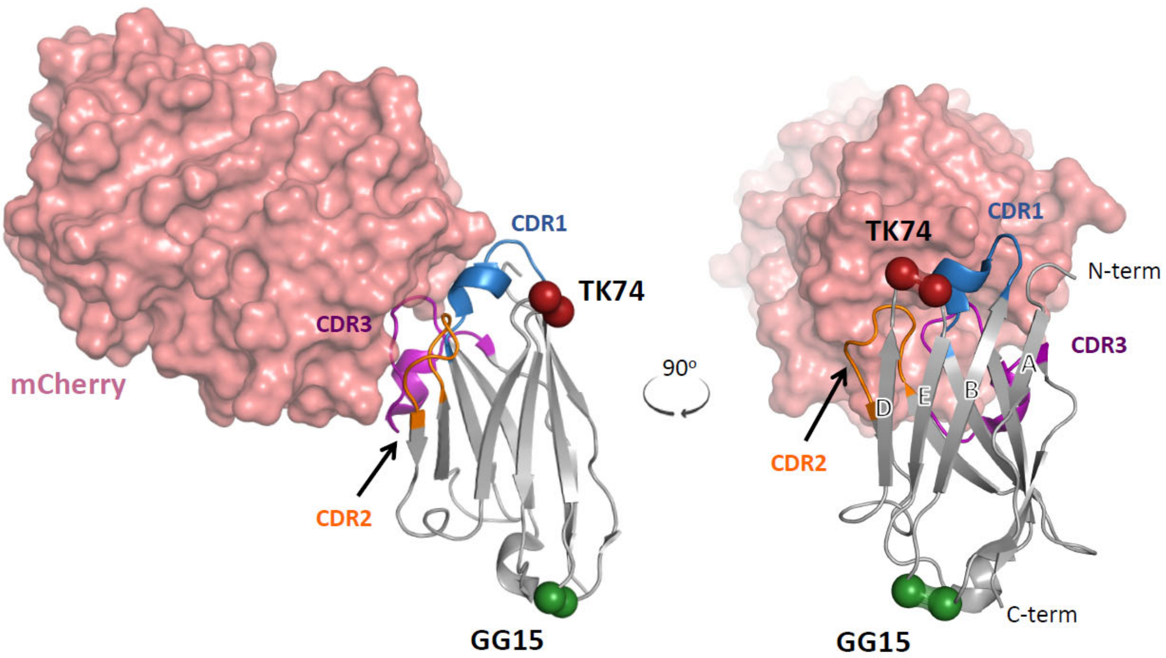

Supplementary Figure 3: Crystal structure of the LaM4 nanobody in complex with its target mCherry (PDB: 6IR1). The structure of LaM4 is shown as a cartoon, while the structure of mCherry is represented as a surface. The positions of the TK74 (red balls) and GG15 (green balls) insertion sites are indicated. CDRs are colored as previously described in Fig 1. Two views are presented, rotated 90 degrees around the y-axis.

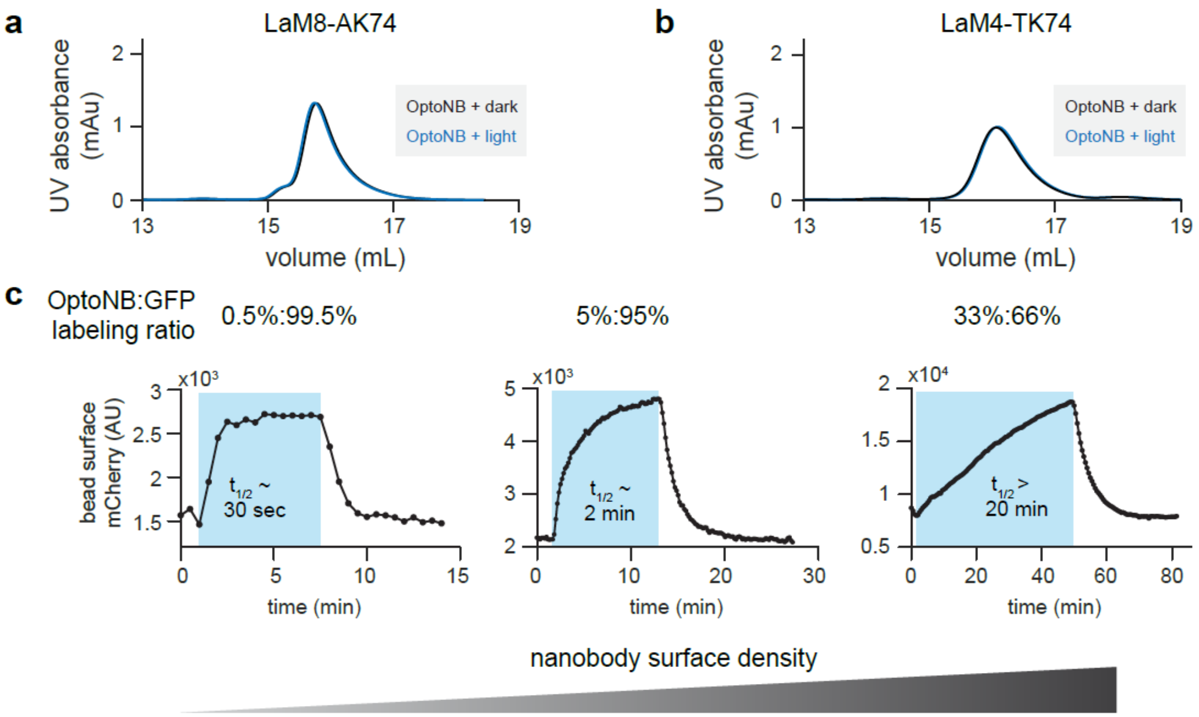

Supplementary Figure 4: In vitro characterization of OptoNBs. (**a-b**) SEC elution profile for LaM8-AK74 (in **a**) and LaM4 TK74 (in **b**). Dark- and light-incubated OptoNB are shown in the indicated curves. (**c**) Quantification of mCherry binding to LaM8-AK74 coated beads. His-tagged LaM8-AK74 OptoNBs and His-tagged eGFP were immobilized on the surface of NiNTA agarose beads in various ratios (0.5%:99.5%; 5%:95%; 33%:66%). Each was incubated in a solution of 1 µM mCherry, and mCherry intensity on the bead surface was quantified over time. Increasing the nanobody surface density resulted in slower mCherry binding/unbinding kinetics as well as a shift from exponential to linear kinetics, as predicted from the formation of a diffusion-limited mCherry depletion layer in the vicinity of the bead.

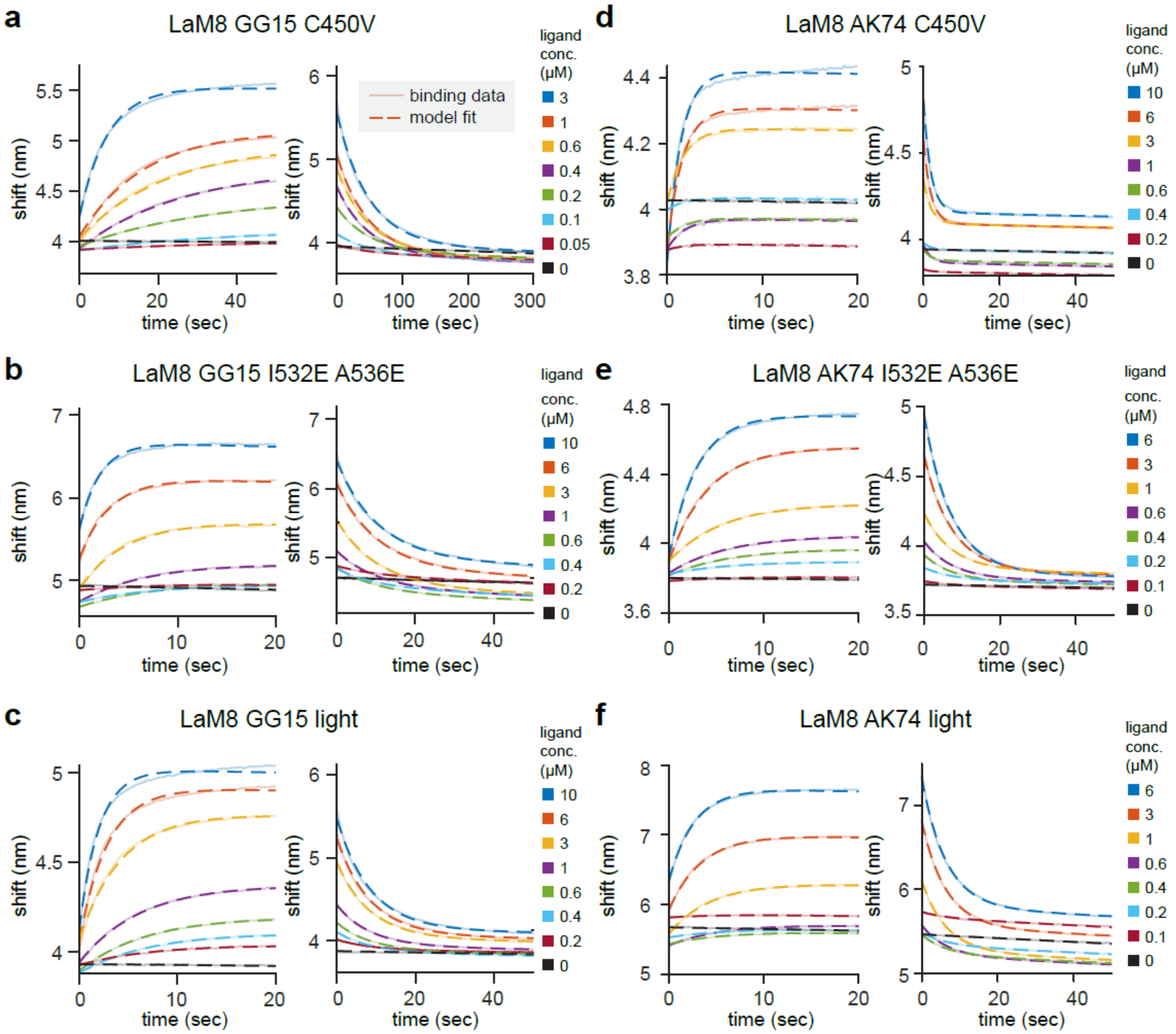

Supplementary Figure 5: Bio-layer interferometry (BLI) to measure opto-nanobody binding kinetics. (**a-f**) BLI traces for LaM8 GG15 (in **a-c**) and LaM8 AK74 (in **d-f**). All plots show the raw BLI shifts for immobilized OptoNB variants over time in various concentrations of mCherry. Raw data are shown as solid curves; model fits are shown in dashed curves. To measure the binding constants from the dark/pre-illuminated state, we used a dark-like C450V mutant that is unable to transduce light absorption into a conformational change (in **a** and **d**). For lit-state measurements we compared lit-like I532E/A536E double mutant (in **b** and **e**) to a true lit-state produced by illuminating the BLI sensors with a constant 450 nm LED light input (in **c** and **f**). The corresponding values of k_{on}, k_{off}, and K_D are shown in **Table 1**.

Supplementary Table 1: In-cell binding results for the opto-nanobody against actin

Insertion site	none	GG 15	AK 74	GG 15	AK 74	GS 16	KE 43	A40-E44	P41-E44	D62-E66	F63-G66	DN 72	NA 73	KN 75
LOV variant	none	s	s	o	o	o	o	o	o	o	o	o	o	o
Actin binding	Y	N	N	N	N	N	Y	Y	Y	Y	Y	N	N	N
Photo-switching	N	N	N	N	N	N	N	N	N	Y	N	N	N	N

Y: binding or photoswitching observed. N: binding or photoswitching not observed.

Supplementary Note 1

LaM8-AK74 with sLOV insertion (black: nanobody; blue: LOV):

```
atggcccaagtgcagctggtagagagtggcggcggcttggtccaggcaggtggaagtttgcggcttagct
gcgctgtgagtgggcgacctttttcagagtacaacctgggatggtttcggcaggcccccggtaaagaacg
ggagttcgttgcccgcatcagatccagcgggactaccgtgtataccgactccgtcaagggtagattctct
gcaagtagggacaacgctGGTTTGGAACGTATCGAAAAGAATTTTGTCATCACGGATCCGCGTCTTCCCG
ACAATCCGATTATCTTCGCGTCAGACTCTTTCTTACAACTGACTGAGTATAGTAGAGAGGAGATATTGGG
GCGTAACTGTAGATTTCTTCAGGGGCCAGAAACTGATCGGGCTACCGTTCGCAAGATACGTGACGCAATA
GACAACCAGACCGAGGTGACGGTGCAGCTGATTAACTACACAAAGTCTGGGAAGAAGTTCTGGAACCTGT
TTCATTTACAACCTATGAGAGACCAAAAAGGTGACGTTCAATATTTCATCGGGGTTCAGTTAGATGGGAC
TGAGCACGTGAGAGATGCAGCAGAAAGAGAGGGTGTAATGCTTATTAAAAAAACAGCCGAGAATATCGAC
GAAGCCGCTGGTaaaaacatggggtatctccagcttaatagtctggagcccgaggataccgccgtatatt
actgtgccatgtccagggtcgatactgattctcctgccttttatgactattggggtcaggggacccaggt
tactgtatcaacaccccggtca
```

LaM8-GG15 with sLOV insertion (black: nanobody; blue: LOV):

```
atggcccaagtgcagctggtagagagtggcggcggcttggtccaggcaggtGGTTTGGAACGTATCGAAA
AGAATTTTGTCATCACGGATCCGCGTCTTCCCGACAATCCGATTATCTTCGCGTCAGACTCTTTCTTACA
ACTGACTGAGTATAGTAGAGAGGAGATATTGGGGCGTAACTGTAGATTTCTTCAGGGGCCAGAAACTGAT
CGGGCTACCGTTCGCAAGATACGTGACGCAATAGACAACCAGACCGAGGTGACGGTGCAGCTGATTAACT
ACACAAAGTCTGGGAAGAAGTTCTGGAACCTGTTTCATTTACAACCTATGAGAGACCAAAAAGGTGACGT
TCAATATTTCATCGGGGTTCAGTTAGATGGGACTGAGCACGTGAGAGATGCAGCAGAAAGAGAGGGTGTA
ATGCTTATTAAAAAAACAGCCGAGAATATCGACGAAGCCGCTGGTggaagtttgcggcttagctgcgctg
tgagtgggcgacctttttcagagtacaacctgggatggtttcggcaggcccccggtaaagaacgggagtt
cgttgcccgcatcagatccagcgggactaccgtgtataccgactccgtcaagggtagattctctgcaagt
agggacaacgctaaaaacatggggtatctccagcttaatagtctggagcccgaggataccgccgtatatt
actgtgccatgtccagggtcgatactgattctcctgccttttatgactattggggtcaggggacccaggt
tactgtatcaacaccccggtca
```

Actin OptoNB with LOV insertion (black: nanobody; blue: LOV):

ATGGCTCAGGTGCAGCTGGTGGAGTCTGGGGGAGGACTGACGCAGGCAGGGGGCTCTCTGAGACTCTCCT
GTGCAACCTCTGGACTAATCTTCAGTGCCTTTGGCATGGGCTGGTTCCGCCAGGCTCCAGGGAAGGAGCG
TGAGTTTGTAGGAGGTATTAACTGGAGGGGTAGTACAAACTATGGAGACTTGGCTACTACACTTGAACGT
ATTGAGAAGAACTTTGTCATTACTGACCCAAGATTGCCAGATAATCCCATTATATTCGCGTCCGATAGTT
TCTTGCAGTTGACAGAATATAGCCGTGAAGAAATTTTGGGAAGAAACTGCAGGTTTCTACAAGGTCCTGA
AACTGATCGCGCGACAGTGAGAAAAATTAGAGATGCCATAGATAACCAAACAGAGGTCACTGTTCAGCTG
ATTAATTATACAAAGAGTGGTAAAAAGTTCTGGAACCTCTTTCACTTGCAGCCTATGCGAGATCAGAAGG
GAGATGTCCAGTACTTTATTGGGGTTCAGTTGGATGGAACTGAGCATGTCCGAGATGCTGCCGAGAGAGA
GGGAGTCATGCTGATTAAGAAAACTGCAGAAAATATTGATGAGGCGGCAAAAGAACTTGGCCGATTCACC
ATCTCCAGAGACAACGCCAAGAACACGGTGTATTTGCAGATGAACAACCTGAAACCTGAGGACACGGCCG
TTTATTACTGCGCAGCACGTATGGTTCACAAAACCGAGTATGACTATTGGGGCGAGGGGACCCAGGTCAC
CGTCTCCTCAAGAAGCTT
