# Supplementary material for: Optogenetic control of protein binding using light-switchable nanobodies
Source: Nat Commun. 2020 Aug 13;11:4044. doi: 10.1038/s41467-020-17836-8 (PMC7426870; doi:10.1038/s41467-020-17836-8)
Supplement: Supplementary file 2 — Description of Additional Supplementary Files [file 41467_2020_17836_MOESM2_ESM.pdf]

Description of Additional Supplementary Files

File Name: Supplementary Movie 1
Description: Time-lapse imaging of HEK293T cells expressing the LaM8-GG15_irFP OptoNB (shown) and membrane-localized mCherry-CAAX (not shown). Cells were imaged using a 60X oil objective every 20 sec for 30 min as 450 nm blue LED illumination was toggled on and off (indicated by the blue box in the lower-left corner). Timer indicates mm:ss; scale bar indicates 10 µm.

File Name: Supplementary Movie 2
Description: Time-lapse imaging of HEK293T cells expressing the LaM8-AK74_irFP OptoNB (shown) and membrane-localized mCherry-CAAX (not shown). Cells were imaged using a 60X oil objective every 20 sec for 30 min as 450 nm blue LED illumination was toggled on and off (indicated by the blue box in the lower-left corner). Timer indicates mm:ss; scale bar indicates 10 µm.

File Name: Supplementary Movie 3
Description: Time-lapse imaging of an NIH3T3 cell expressing LaM8-GG15_SOScat OptoNB fusion protein (not shown), membrane-localized mCherry-CAAX (not shown), and ErkKTR-irFP (shown). Cells were imaged using a 60X oil objective every 30 sec for 80 min as 450 nm blue LED illumination was toggled on and off (indicated by the blue box in the lower-left corner). Timer indicates hh:mm; scale bar indicates 10 µm.

File Name: Supplementary Movie 4
Description: Time-lapse imaging of an NIH3T3 cell expressing LaM8-AK74_SOScat OptoNB fusion protein (not shown), membrane-localized mCherry-CAAX (not shown), and ErkKTR-irFP (shown). Cells were imaged using a 60X oil objective every 30 sec for 80 min as 450 nm blue LED illumination was toggled on and off (indicated by the blue box in the lower-left corner). Timer indicates hh:mm; scale bar indicates 10 µm.

File Name: Supplementary Movie 5
Description: Time-lapse imaging of a NiNTA-coated agarose bead with His-tagged LaM8-GG15 and His-tagged GFP immobilized on its surface in a 0.1%:99.9% ratio, in a solution of 2 µM untagged, soluble mCherry. The bead was imaged using a 20X air objective every 30 sec for 30 min as 450 nm blue LED illumination was toggled on and off (indicated by the blue box in the lower-left corner). Timer indicates hh:mm; scale bar indicates 50 µm.

File Name: Supplementary Movie 6
Description: Time-lapse imaging of a NiNTA-coated agarose bead with His-tagged LaM8-AK74 and His-tagged GFP immobilized on its surface in a 5%:95% ratio, in a solution of 1 µM untagged, soluble mCherry. The bead was imaged using a 20X air objective every 30 sec for 80 min as 450 nm blue LED illumination was toggled on and off (indicated by the blue box in the lower-left corner). Timer indicates hh:mm; scale bar indicates 50 µm.

File Name: Supplementary Movie 7
Description: Time-lapse imaging of an NIH3T3 cell expressing OptoNB(actin)-TagRFP. Cells were imaged using a 60X oil objective every 20 sec for 60 min, and 450 nm blue LED illumination was toggled on and off (indicated by the blue box in the upper-right corner). Timer indicates hh:mm; scale bar indicates 20 µm.

File Name: Supplementary Movie 8
Description: Time-lapse imaging of an NIH3T3 cell expressing OptoNB(actin)-TagRFP. Cells were imaged using a 60X oil objective every 20 sec for 60 min, and 450 nm blue LED illumination was moved from the right half of the cell to the left half (indicated by the position of the blue bar above the cell). Timer indicates hh:mm; scale bar indicates 20 µm.
