# Supplementary material for: Optogenetic control of protein binding using light-switchable nanobodies
Source: Nat Commun. 2020 Aug 13;11:4044. doi: 10.1038/s41467-020-17836-8 (PMC7426870; doi:10.1038/s41467-020-17836-8)
Supplement: Supplementary file 11 — Reporting Summary [file 41467_2020_17836_MOESM11_ESM.pdf]

nature research

Corresponding author(s): Toettcher JE & Avalos JL

Last updated by author(s): 7/6/2020

Reporting Summary

Nature Research wishes to improve the reproducibility of the work that we publish. This form provides structure for consistency and transparency in reporting. For further information on Nature Research policies, see our Editorial Policies and the Editorial Policy Checklist.

Statistics

For all statistical analyses, confirm that the following items are present in the figure legend, table legend, main text, or Methods section.

n/a	Confirmed	
☐	☒	The exact sample size (*n*) for each experimental group/condition, given as a discrete number and unit of measurement
☒	☐	A statement on whether measurements were taken from distinct samples or whether the same sample was measured repeatedly
☐	☒	The statistical test(s) used AND whether they are one- or two-sided<br>*Only common tests should be described solely by name; describe more complex techniques in the Methods section.*
☒	☐	A description of all covariates tested
☒	☐	A description of any assumptions or corrections, such as tests of normality and adjustment for multiple comparisons
☐	☒	A full description of the statistical parameters including central tendency (e.g. means) or other basic estimates (e.g. regression coefficient) AND variation (e.g. standard deviation) or associated estimates of uncertainty (e.g. confidence intervals)
☐	☒	For null hypothesis testing, the test statistic (e.g. *F*, *t*, *r*) with confidence intervals, effect sizes, degrees of freedom and *P* value noted<br>*Give P values as exact values whenever suitable.*
☒	☐	For Bayesian analysis, information on the choice of priors and Markov chain Monte Carlo settings
☒	☐	For hierarchical and complex designs, identification of the appropriate level for tests and full reporting of outcomes
☒	☐	Estimates of effect sizes (e.g. Cohen's *d*, Pearson's *r*), indicating how they were calculated

Our web collection on statistics for biologists contains articles on many of the points above.

Software and code

Policy information about availability of computer code

Data collection	All microscopy data was collected using NIS Elements version 4.4 (Nikon).
Data analysis	All data was quantified in ImageJ v1.52p, and analyzed using custom MATLAB scripts.<br>See the paper's Github repository for all code at: https://github.com/toettchlab/Gil2020, which is linked in the Methods section.

For manuscripts utilizing custom algorithms or software that are central to the research but not yet described in published literature, software must be made available to editors and reviewers. We strongly encourage code deposition in a community repository (e.g. GitHub). See the Nature Research guidelines for submitting code & software for further information.

Data

Policy information about availability of data

All manuscripts must include a data availability statement. This statement should provide the following information, where applicable:

- Accession codes, unique identifiers, or web links for publicly available datasets
- A list of figures that have associated raw data
- A description of any restrictions on data availability

The data that support the findings of this study are available from the corresponding author upon reasonable request. Raw data for Figures 1D, 2B-D, 3B-D, 4C-F, 5C-E, S1C, S2A, S4C, and Table 1 are provided in an accompanying file.

Field-specific reporting

Please select the one below that is the best fit for your research. If you are not sure, read the appropriate sections before making your selection.

☒ Life sciences ☐ Behavioural & social sciences ☐ Ecological, evolutionary & environmental sciences

For a reference copy of the document with all sections, see nature.com/documents/nr-reporting-summary-flat.pdf

Life sciences study design

All studies must disclose on these points even when the disclosure is negative.

Sample size	Sample sizes were chosen based on maximizing data given the constraints of live-cell imaging (we needed objective lens magnification of ~60X to assess light-induced protein movement while still attempting to collect as many cells as possible per field of view). This led to typical sample sizes of n=5-10 cells per experiment. Nevertheless, cellular responses were sufficiently robust to obtain clear results for light-induced translocation.
Data exclusions	None.
Replication	All major findings were repeated at least three times to ensure reproducibility, and all attempts at reproduction were successful. One exception to this general rule was the initial screens for light-switchable nanobodies were sufficiently large-scale to preclude triplicate repeats at all insertion sites (most of which were negative for light-switchable responses). However, the majority of the paper's subsequent experiments were done to validate these AK74 and GG15 hits.
Randomization	Sample randomization was not performed because the authors had no prior expectations or biases for which LOV domain insertions would generate photoswitchable nanobody binding, so there was no incentive to assign responses to a particular construct.
Blinding	Author blinding to specific nanobody variants was not performed because the authors had no prior expectations or biases for which LOV domain insertions should generate photoswitchable nanobody binding, so there was no incentive to assign responses to a particular construct.

Reporting for specific materials, systems and methods

We require information from authors about some types of materials, experimental systems and methods used in many studies. Here, indicate whether each material, system or method listed is relevant to your study. If you are not sure if a list item applies to your research, read the appropriate section before selecting a response.

Materials & experimental systems

n/a	Involved in the study	
☐	☒	Antibodies
☐	☒	Eukaryotic cell lines
☒	☐	Palaeontology and archaeology
☒	☐	Animals and other organisms
☒	☐	Human research participants
☒	☐	Clinical data
☒	☐	Dual use research of concern

Methods

n/a	Involved in the study	
☒	☐	ChIP-seq
☒	☐	Flow cytometry
☒	☐	MRI-based neuroimaging

Antibodies

Antibodies used	DNA constructs encoding the anti-actin "chromobody" (from Chromotek), and LaM4/LaM8/LaG4 nanobodies were modified and expressed in cells (Fridy et al, Nat Meth 2014).
Validation	Sequences were validated against published sequences for each nanobody used. All nanobodies were expressed and/or purified in the lab and clearly bound to their intended targets (GFP, mCherry and actin) in our assays.

Eukaryotic cell lines

Policy information about cell lines

Cell line source(s)	NIH3T3 cells, HEK293 cells were purchased from ATCC.
Authentication	Cell lines were not authenticated beyond what was performed by ATCC.

April 2020

Mycoplasma contamination	Cell lines are periodically tested for mycoplasma (approx. once per year) and only negative-testing cell lines are used in the lab.
Commonly misidentified lines<br>(See ICLAC register)	No commonly misidentified cell lines were used in this study.
